# Perceived knowledge, attitudes, and practices regarding nutritional management among gastric cancer patients: an exploratory path analysis

**DOI:** 10.3389/fnut.2026.1766768

**Published:** 2026-08-05

**Authors:** Jun Ma, Fei Gao, Guolong Ma, Jing Zhang, Xiaoyuan Qiao

**Affiliations:** 1 Department of General Surgery, Cancer Hospital Affiliated to Shanxi Medical University, Shanxi Province Cancer Hospital, Shanxi Hospital Affiliated to Cancer Hospital, Chinese Academy of Medical Sciences, Taiyuan, Shanxi, China; 2 Department of Comprehensive Medicine, Cancer Hospital Affiliated to Shanxi Medical University, Shanxi Province Cancer Hospital, Shanxi Hospital Affiliated to Cancer Hospital, Chinese Academy of Medical Sciences, Taiyuan, Shanxi, China

**Keywords:** attitudes, gastric cancer, knowledge, nutritional management, practices

## Abstract

**Objectives:**

This cross-sectional study aimed to evaluate the perceived knowledge, attitudes, and practices (KAP) regarding nutritional management among patients with gastric cancer (GC).

**Methods:**

The study was conducted among patients with histopathologically confirmed GC at Shanxi Provincial Cancer Hospital between April 2025 and September 2025. Participants completed a self-administered questionnaire capturing socio-demographic characteristics and KAP scores. Exploratory path analysis was employed to examine interrelationships among the KAP domains.

**Results:**

A total of 828 valid questionnaires were analyzed, yielding an effective response rate of 98.8%. Mean scores for perceived knowledge, attitudes, and practices were 16.48 ± 3.96 (range: 0–26), 44.19 ± 2.06 (range: 10–50), and 23.58 ± 1.86 (range: 6–30), respectively. KAP scores varied significantly across subgroups defined by education level, area of residence, and prior receipt of nutritional education (all *P* < 0.001). Knowledge was positively correlated with attitudes (*r* = 0.284, *P* < 0.001) and practices (*r* = 0.163, *P* < 0.001), and attitudes were positively correlated with practices (*r* = 0.230, *P* < 0.001). Path analysis revealed that perceived knowledge was directly associated with attitudes (β = 0.417, 95% CI: 0.311–0.490, *P* = 0.019) and demonstrated a significant total association with practices (β = 0.160, 95% CI: 0.076–0.241, *P* = 0.033). Attitudes were directly associated with practices (β = 0.311, *P* = 0.007) and served as a potential mediator in the relationship between perceived knowledge and practices (indirect β = 0.130, *P* = 0.004).

**Conclusion:**

Despite generally positive attitudes, substantial gaps remain in GC patients’ nutritional knowledge and implementation of healthy practices. Structured, skill-based nutritional education, integrated within a multidisciplinary care framework, may enhance adherence.

## Introduction

Gastric cancer (GC) remains one of the most prevalent and lethal malignancies worldwide, ranking fifth in incidence and fourth in cancer-related mortality globally, with an estimated 1.09 million new cases and 770,000 deaths in 2020 (1). The burden is particularly severe in East Asia—China alone accounts for nearly 44% of global GC cases and 48% of deaths, making it a major public health concern (2). Given the substantial global and regional disease burden, understanding and addressing challenges in GC patients has become increasingly critical.

Recent clinical and translational researches have driven significant advances in the diagnostic and therapeutic landscape of GC. For instance, deeper insights into the heterogeneous tumor microenvironment (TME), particularly the crosstalk between cancer-associated fibroblasts and myeloid cells, have revealed new metabolic and immunosuppressive mechanisms (3). Consequently, novel therapeutic agents, including multi-target pharmacological compounds like P-hydroxylcinnamaldehyde (4), anti-cancer bioactive peptides mediating the TP53 apoptotic cascade (5), and other emerging targeted approaches (6), are being extensively investigated to improve survival outcomes. However, the successful implementation and tolerance of these advanced treatments heavily rely on patients’ physical resilience. Despite advances in diagnostic and therapeutic approaches, a large proportion of GC patients experience malnutrition or nutritional risk, with studies reporting prevalence rates ranging from 38.9% to 75% depending on the assessment tool and disease stage (7, 8). A multicenter cohort study in China revealed that more than half (51.1%) of GC patients were at nutritional risk, and those with high-risk scores (NRS2002 ≥ 5) had significantly higher rates of postoperative infections (7). Such nutritional deterioration contributes to impaired treatment tolerance, delayed recovery, and decreased overall survival. A meta-analysis found that malnutrition doubled the risk of mortality among patients with gastrointestinal cancers (8). Therefore, nutritional management plays a vital role in the comprehensive care of GC patients. Adequate nutrition supports immune function, enhances treatment tolerance, reduces postoperative complications, and improves quality of life. Evidence from clinical studies demonstrates that structured nutritional interventions, such as dietary counseling, oral nutritional supplements, and enteral or parenteral nutrition, can significantly improve outcomes (9, 10).

Knowledge, attitudes, and practices (KAP) studies, widely used in public health, evaluate what people know, how they feel, and how they act regarding a specific topic. In the context of cancer nutrition, KAP research helps identify misconceptions, behavioral barriers, and education needs that hinder effective nutritional management. Although nutritional care has gained increasing attention in oncology, existing KAP studies have mainly focused on other cancer populations. For example, women with breast cancer also demonstrated suboptimal nutritional knowledge, attitudes and practices in Iran (11). A 2025 scoping review of nutrition-exercise KAP in cancer survivors reported a large body of work across cancer sites, but noted the relative paucity of research in GC populations (12). However, evidence specific to GC patients remains scarce, despite their uniquely high nutritional risk due to tumor-related obstruction, decreased intake, malabsorption, and treatment-related gastrointestinal symptoms.

Given these challenges, a systematic evaluation of patients’ KAP toward nutritional management is urgently needed. This study aims to assess the current status of knowledge, attitudes, and practices regarding nutritional management among GC patients.

## Materials and methods

### Study design and participants

This cross-sectional study was conducted between April 2025 and September 2025 at Shanxi Provincial Cancer Hospital. Participants were patients with histopathologically confirmed GC diagnosed according to the *WHO Classification of Tumors of the Digestive System* (2019) (13). The study protocol was approved by the Institutional Review Board of Shanxi Provincial Cancer Hospital (Approval No. KY2025029). The inclusion criteria were as follows: (1) Age 18 years or older; (2) A histopathologically confirmed diagnosis of GC; (3) Being scheduled for or having undergone initial surgical resection for GC, which was used to define a clinically relevant surgical-care population while allowing inclusion of patients at different stages of the treatment trajectory; (4) Awareness of their actual diagnosis and willingness to participate in the study interviews. Patients were excluded if they had severe cognitive impairment, were unable to complete a self-administered questionnaire, suffered from severe intercurrent illnesses, or had a concurrent second malignancy.

### Questionnaire

The questionnaire was developed based on relevant clinical guidelines on nutritional management for GC, including the *Expert consensus on nutritional treatment for patients with gastric cancer* (14), and the *Nutrition Treatment Guidelines for Gastric Cancer Patients* (15). To ensure content validity, a panel of 12 multidisciplinary experts—including gastrointestinal surgeons, oncologists, clinical nutritionists, and oncology nurses, each with at least 10 years of professional experience—was invited to conduct expert consultations. According to expert feedback, the wording of knowledge and attitude items was refined for clarity, while practice items were operationalized into measurable behaviors such as frequency of self-weighing and dietary monitoring. A pilot test among 29 patients demonstrated excellent internal consistency (Cronbach’s α = 0.921 overall; 0.891 for knowledge, 0.777 for attitudes, and 0.847 for practices). Confirmatory factor analysis (CFA) further supported the structural validity of the scale, showing acceptable model fit indices (Chi-square minimum discrepancy divided by degrees of freedom (CMIN/DF) = 3.264, root mean square error of approximation (RMSEA) = 0.052, incremental fit index (IFI) = 0.849, Tucker–Lewis index (TLI) = 0.806, comparative fit index (CFI) = 0.847) (Supplementary Figure 1), and the detailed item-level standardized factor loadings are presented in Supplementary Table 2. In the full study cohort, the overall questionnaire demonstrated acceptable internal consistency (α = 0.773). In addition, corrected item-total correlations and Cronbach’s α-if-item-deleted values were calculated at the overall questionnaire level (Supplementary Table 3).

The finalized Chinese questionnaire (provided in full as Supplementary material, along with the English translation) comprises four sections. The first section assesses sociodemographic and clinical characteristics, including age, sex, education level, residence, monthly income, anthropometric measures, time since gastric cancer diagnosis, family history, treatment modalities, lifestyle factors, dietary habits, sleep quality, and previous receipt of nutrition-related education or counseling. The perceived knowledge domain contains 13 items scored as two points for “well-understood,” 1 point for “heard of,” and 0 points for “unclear,” with total scores ranging from 0 to 26. The attitudes domain includes 10 items rated on a five-point Likert scale from “strongly agree” to “strongly disagree,” resulting in a possible score of 10–50. Items 9 and 10 in the attitudes domain represent perceived burdens and were reverse-scored, while all other items were forward-scored. The practices domain consists of 8 items, of which items 1–6 are scored on a five-point frequency scale from “never” to “always” (total score: 6–30), while items P7 and P8 are open-ended questions exploring barriers to nutritional management and sources of nutritional information. These two items are not scored and are intended for descriptive analysis only. According to Bloom’s cut-off, which is widely adopted in KAP studies, KAP levels were classified as poor (<60%), moderate (60%–79%), or good (≥80%) (16).

### Questionnaire distribution and quality control

Data collection followed a convenience sampling approach. Questionnaires were distributed both online via the WJX platform (Questionnaire Star^1^) and in person by trained investigators. The survey’s QR code was shared through WeChat to facilitate access. Investigators supervised on-site data collection to clarify items and ensure completeness. To maintain data integrity, a dual-entry system was implemented, and questionnaires were excluded if they failed a common-sense attention-check trap item (“The Han ethnic group has the smallest population among China’s 56 ethnic groups”), contained implausible demographic information (e.g., age 150 years), showed repetitive or uniform responses, had substantial missing data, or were completed in less than 90 s. These predefined quantitative rules were strictly applied to exclude invalid responses and ensure data reliability.

### Sample size

The sample size was estimated using the standard single-population proportion formula commonly applied in KAP studies:


$$n={\left(\left(Z\,\_\,\left(1-\alpha /2\right)\right)/\delta \right)}^{\wedge }\,2\times p\,\left(1-p\right)$$

A 95% confidence level [Z_(1−α/2) = 1.96], with a margin of error (d) of 0.05 and an assumed proportion (p) of 0.50 in the absence of prior KAP data for this topic, the minimum required sample size was:


$$n={\left(1.96/0.05\right)}^{\wedge }\,2\times 0.50\times 0.50\times 384$$

To allow for approximately 20% invalid or incomplete responses, we targeted a sample size of at least 480 participants.

### Statistical analysis

All statistical analyses were performed using SPSS 26.0 and AMOS 26.0 (IBM, Armonk, NY, USA). The Shapiro–Wilk test was applied to assess normality of distribution. Continuous variables were expressed as mean ± standard deviation. Group comparisons were conducted using independent-samples *t*-tests or Mann–Whitney U tests for two groups and one-way ANOVA or Kruskal–Wallis tests for three or more groups. Correlations among knowledge, attitude, and practice scores were analyzed using Spearman’s coefficients. Exploratory path analysis was then employed to test the hypothesized mediating role of attitudes between perceived knowledge and practices based on the KAP theoretical framework. Prior to path analysis, the three domain scores were examined for distributional properties. Given the disparate raw score ranges across domains, standardized path coefficients (β) were reported to ensure comparability and eliminate bias from raw scale variance. Path analysis was conducted using maximum likelihood (ML) estimation in AMOS 26.0. Multivariate normality was confirmed (Mardia’s multivariate kurtosis c.r. < 5). Because the model with three observed variables and three directional paths was saturated (df = 0), conventional fit indices were not applicable. Standardized direct, indirect, and total associations with 95% bias-corrected confidence intervals (CIs) were estimated using bootstrap resampling (5,000 iterations) with the bias-corrected percentile method. A two-tailed *p*-value < 0.05 was considered statistically significant.

## Results

A total of 838 questionnaires were collected. After excluding one participant under 18 years of age, one with anomalous data, three with incomplete information, one that failed the attention-check question, and four completed in less than 90 s, 828 valid responses were included in the final analysis (effective response rate: 98.8%) (Supplementary Figure 2).

### Demographic characteristics and overall KAP scores

Demographic characteristics and KAP scores are summarized in Table 1. The majority of patients were male (59.18%), older than 60 years (56.88%), living in urban areas (55.68%), married (81.52%), diagnosed at stage II–III (74.64%), 3–6 months post-diagnosis (38.53%), without a family history of gastric cancer (65.58%), and having undergone surgical treatment (90.94%). The mean knowledge, attitude, and practice scores were 16.48 ± 3.96 (possible range: 0–26), 44.19 ± 2.06 (possible range: 10–50), and 23.58 ± 1.86 (possible range: 6–30), respectively. Across all three KAP domains, gastric cancer stage, time since diagnosis, dietary habits, and surgical status emerged as the key factors consistently influencing patients’ nutritional knowledge, attitudes, and practices (all *P* < 0.05). To demonstrate the clinical significance of these subgroup differences, effect sizes (Cohen’s d and partial η^2^p) were calculated (Table 1).

**TABLE 1:** Characteristics of gastric cancer patients and comparison of knowledge, attitudes, and practices (KAP) scores across demographic subgroups (*n* = 828).

Variables	*N* (%)	Knowledge (range: 0–26)			Attitude (range: 10–50)			Practice (range: 6–30)		
		Mean ± SD	*t/F, P*	Effect size	Mean ± SD	*t/F, P*	Effect size	Mean ± SD	*t/F, P*	Effect size
Total	828 (100.00)	16.48 ± 3.96			44.19 ± 2.06			23.58 ± 1.86		
Gender			*t* = 0.416, *P* = 0.677	d = 0.029 (neg.)		*t* = −2.306, *P* = 0.021	d = −0.160 (neg.)		*t* = −0.155, *P* = 0.877	d = −0.011 (neg.)
Male	490 (59.18)	16.52 ± 3.99	–	–	44.06 ± 2.13	–	–	23.57 ± 1.89	–	–
Female	338 (40.82)	16.41 ± 3.92	–	–	44.38 ± 1.94	–	–	23.59 ± 1.83	–	–
Age (years)			*t* = −1.769, P = 0.077	d = −0.122 (neg.)		*t* = 1.898, *P* = 0.058	d = 0.132 (neg.)		*t* = 0.968, *P* = 0.333	d = 0.069 (neg.)
≤60	357 (43.12)	16.20 ± 3.72	–	–	44.34 ± 2.01	–	–	23.65 ± 1.97	–	–
>60	471 (56.88)	16.69 ± 4.12	–	–	44.07 ± 2.09	–	–	23.52 ± 1.78	–	–
BMI (kg/m^2^)			*t* = −1.545, *P* = 0.123	d = −0.107 (neg.)		*t* = −2.180, *P* = 0.030	d = −0.152 (neg.)		*t* = 0.265, *P* = 0.791	d = 0.018 (neg.)
Underweight/normal (≤23.9)	419 (50.60)	16.27 ± 4.12	–	–	44.04 ± 2.06	–	–	23.60 ± 1.90	–	–
Overweight/obese (>23.9)	409 (49.40)	16.69 ± 3.79	–	–	44.35 ± 2.05	–	–	23.56 ± 1.83	–	–
Residential area			*t* = −3.959, *P* < 0.001	d = −0.285 (small)		*t* = −3.654, *P* < 0.001	d = −0.265 (small)		t = −3.369, *P* < 0.001	d = −0.235 (small)
Rural	367 (44.32)	15.86 ± 4.42	–	–	43.89 ± 2.38	–	–	23.34 ± 1.81	–	–
Urban	461 (55.68)	16.97 ± 3.48	–	–	44.43 ± 1.73	–	–	23.77 ± 1.88	–	–
Marital status	–	–	*t* = −2.540, *P* = 0.012	d = −0.260 (small)	–	*t* = −3.399, *P* < 0.001	d = −0.361 (small)	–	*t* = −4.978, *P* < 0.001	d = −0.507 (medium)
Unmarried	153 (18.48)	15.64 ± 4.67	–	–	43.59 ± 2.52	–	–	22.82 ± 2.15	–	–
Married	675 (81.52)	16.67 ± 3.76	–	–	44.33 ± 1.92	–	–	23.75 ± 1.75	–	–
Total	828 (100.00)	16.48 ± 3.96			44.19 ± 2.06			23.58 ± 1.86		
Education level			*F* = 18.323, *P* < 0.001	η^2^p = 0.043 (small)		*F* = 1.512, *P* = 0.221	η^2^p = 0.004 (neg.)		*F* = 35.525, *P* < 0.001	η^2^p = 0.079 (medium)
Junior high or below	420 (50.72)	16.35 ± 4.14	–	–	44.08 ± 2.16	–	–	23.45 ± 1.76	–	–
Senior high/vocational	301 (36.35)	17.29 ± 3.15	–	–	44.25 ± 1.88	–	–	23.28 ± 1.83	–	–
College or above	107 (12.92)	14.68 ± 4.61	–	–	44.44 ± 2.13	–	–	24.93 ± 1.78	–	–
Monthly income (RMB)			*F* = 26.811, *P* < 0.001	η^2^p = 0.061 (medium)		*F* = 1.712, *P* = 0.181	η^2^p = 0.004 (neg.)		*F* = 0.456, *P* = 0.634	η^2^p = 0.001 (neg.)
<2,000	279 (33.70)	15.46 ± 4.50	–	–	44.12 ± 2.20	–	–	23.54 ± 1.66	–	–
2,000–5,000	403 (48.67)	17.48 ± 2.89	–	–	44.32 ± 1.77	–	–	23.56 ± 1.81	–	–
>5,000	146 (17.63)	15.65 ± 4.68	–	–	43.97 ± 2.47	–	–	23.71 ± 2.31	–	–
Gastric cancer stage			*F* = 18.935, *P* < 0.001	η^2^p = 0.064 (medium)		*F* = 5.511, *P* < 0.001	η^2^p = 0.020 (small)		*F* = 10.452, *P* < 0.001	η^2^p = 0.037 (small)
I	114 (13.77)	13.98 ± 4.05	–	–	43.68 ± 2.57	–	–	22.98 ± 2.55	–	–
II	312 (37.68)	16.89 ± 2.67	–	–	44.12 ± 1.77	–	–	23.59 ± 1.70	–	–
III	306 (36.96)	16.76 ± 4.25	–	–	44.26 ± 1.96	–	–	23.54 ± 1.63	–	–
IV	96 (11.59)	17.19 ± 5.18	–	–	44.80 ± 2.40	–	–	24.40 ± 1.83	–	–
Time since diagnosis			*F* = 45.925, *P* < 0.001	η^2^p = 0.182 (large)	–	*F* = 4.599, *P* = 0.001	η^2^p = 0.022 (small)	–	*F* = 5.895, *P* < 0.001	η^2^p = 0.028 (small)
<1 month	56 (6.76)	11.41 ± 5.78	–	–	43.61 ± 3.12	–	–	22.64 ± 2.82	–	–
1–3 months	225 (27.17)	15.63 ± 4.08	–	–	44.13 ± 2.18	–	–	23.80 ± 1.87	–	–
3–6 months	319 (38.53)	16.96 ± 3.09	–		44.03 ± 1.89	–	–	23.47 ± 1.63	–	–
6 months–1 year	167 (20.17)	18.51 ± 2.67	–		44.48 ± 1.63	–	–	23.62 ± 1.82	–	–
>1 year	61 (7.37)	16.18 ± 3.72	–	–	44.95 ± 2.09	–	–	24.10 ± 1.71	–	–
Daily exercise time			*F* = 83.669, *P* < 0.001	η^2^p = 0.169 (large)	–	*F* = 0.757, *P* = 0.469	η^2^p = 0.002 (neg.)	–	*F* = 19.128, *P* < 0.001	η^2^p = 0.044 (small)
0 h	329 (39.73)	17.72 ± 2.70	–	–	44.22 ± 2.04	–	–	23.10 ± 1.78	–	–
0–1 h	459 (55.43)	16.14 ± 3.96	–	–	44.20 ± 1.83	–	–	23.90 ± 1.66	–	–
>1 h	40 (4.83)	10.10 ± 5.60	–	–	43.80 ± 3.92	–	–	23.88 ± 3.38	–	–
Sleep status			*t* = −0.715, *P* = 0.475	d = −0.063 (neg.)		*t* = −3.114, *P* = 0.002	d = −0.291 (small)		*t* = −2.765, *P* = 0.006	d = −0.287 (small)
Good	151 (18.24)	16.27 ± 3.89	–	–	43.70 ± 2.15	–	–	23.15 ± 2.21	–	–
Poor/frequent insomnia	677 (81.76)	16.52 ± 3.98	–	–	44.30 ± 2.02	–	–	23.68 ± 1.76	–	–
Family history of gastric cancer			*t* = 4.125, *P* < 0.001	d = 0.290 (small)		*t* = 2.399, *P* = 0.017	d = 0.169 (Neg.)		*t* = −4.811, *P* < 0.001	d = −0.353 (small)
Yes	285 (34.42)	17.22 ± 3.61	–	–	44.42 ± 1.90	–	–	23.15 ± 1.85	–	–
No	543 (65.58)	16.08 ± 4.08	–	–	44.07 ± 2.13	–	–	23.80 ± 1.83	–	–
Smoking			*F* = 1.700, *P* = 0.183	η^2^p = 0.004 (neg.)		*F* = 4.716, *P* = 0.009	η^2^p = 0.011 (small)		*F* = 1.555, *P* = 0.212	η^2^p = 0.004 (neg.)
Never	342 (41.30)	16.43 ± 3.87	–	–	44.45 ± 1.94	–	–	23.60 ± 1.84	–	–
Occasionally	101 (12.20)	17.15 ± 2.84	–	–	44.03 ± 1.82	–	–	23.28 ± 1.92	–	–
Often/heavy	385 (46.50)	16.34 ± 4.27	–	–	44.00 ± 2.20	–	–	23.64 ± 1.86	–	–
Alcohol drinking			*F* = 1.687, *P* = 0.186	η^2^p = 0.004 (neg.)		*F* = 4.813, *P* = 0.008	η^2^p = 0.012 (small)		*F* = 10.220, *P* < 0.001	η^2^p = 0.024 (small)
Never	344 (41.55)	16.58 ± 3.82	–	–	44.41 ± 1.93	–	–	23.63 ± 1.83	–	–
Occasionally	192 (23.19)	16.78 ± 3.92	–	–	43.84 ± 2.12	–	–	23.08 ± 1.71	–	–
Often/Heavy	292 (35.27)	16.15 ± 4.14	–	–	44.15 ± 2.13	–	–	23.85 ± 1.93	–	–
Pre-illness dietary habits			*F* = 18.126, *P* < 0.001	η^2^p = 0.062 (medium)		*F* = 9.899, *P* < 0.001	η^2^p = 0.035 (small)		*F* = 8.854, *P* < 0.001	η^2^p = 0.031 (small)
Light	43 (5.19)	13.65 ± 5.52	–	–	43.60 ± 3.01	–	–	22.93 ± 2.46	–	–
Spicy	103 (12.44)	16.80 ± 4.26	–	–	44.14 ± 1.91	–	–	23.90 ± 1.90	–	–
Greasy	477 (57.61)	17.13 ± 2.77	–	–	44.49 ± 1.77	–	–	23.77 ± 1.72	–	–
Balanced	205 (24.76)	15.39 ± 5.14	–	–	43.63 ± 2.37	–	–	23.12 ± 1.91	–	–
Received nutrition education			*t* = 10.552, *P* < 0.001	d = 0.694 (medium)		*t* = 3.261, *P* = 0.001	d = 0.239 (small)		*t* = 1.148, *P* = 0.251	d = 0.086 (neg.)
Yes	266 (32.13)	18.25 ± 2.88	–	–	44.52 ± 2.00	–	–	23.69 ± 1.87	–	–
No	562 (67.87)	15.64 ± 4.12	–	–	44.03 ± 2.07	–	–	23.53 ± 1.86	–	–
Surgery treatment initiated			*t* = 7.550, *P* < 0.001	d = 1.205 (large)		*t* = 4.284, *P* < 0.001	d = 0.744 (medium)		*t* = 6.015, *P* < 0.001	d = 0.976 (large)
Yes	753 (90.94)	16.89 ± 3.59	–	–	44.33 ± 1.90	–	–	23.74 ± 1.71	–	–
No	75 (9.06)	12.37 ± 5.05	–	–	42.83 ± 2.97	–	–	21.99 ± 2.46	–	–

Continuous KAP scores are reported as Mean ± Standard Deviation (SD). Independent-samples *t*-tests (Welch’s correction) were used for two-group comparisons and one-way ANOVA for multi-group comparisons. Effect sizes: Cohen’s d for two-group comparisons (| d| : 0.2 small, 0.5 medium, 0.8 large); partial η^2^ (η^2^p) for multi-group comparisons (0.01 small, 0.06 medium, 0.14 large; Cohen, 1988).

### Itemized KAP responses

In the perceived knowledge dimension, participants showed generally insufficient understanding of nutrition-related concepts. The three least satisfactory items were: familiarity with the *Dietary Guidelines for Chinese Residents* (only 5.3% were familiar), knowledge of the recommended daily energy intake for GC patients (2.0% reported being well-understood), and awareness that malnutrition may reduce the effectiveness of surgery, radiotherapy, and chemotherapy (20.1% reported being well-understood). In contrast, 77.4% reported understanding that long-term consumption of spicy, pickled, and greasy foods increases GC risk, and 68.5% understood the importance of adequate hydration (Table 2).

**TABLE 2:** Knowledge assessment.

Knowledge items, *n* (%)	Well understood	Heard of	Unclear
1. Gastric cancer patients are highly susceptible to malnutrition	367 (44.32)	406 (49.03)	55 (6.64)
2. Nutritional management is essential for gastric cancer patients throughout the entire treatment course	561 (67.75)	210 (25.36)	57 (6.88)
3. Are you familiar with the Dietary Guidelines for Chinese Residents	44 (5.31)	523 (63.16)	261 (31.52)
4. Gastric cancer patients should maintain a diverse diet and strive for nutritional balance	270 (32.61)	505 (60.99)	53 (6.4)
5. The estimated daily energy requirement for cancer patients is approximately 20-30 kcal per kg of body weight.	17 (2.05)	293 (35.39)	518 (62.56)
6. Compared to healthy individuals, gastric cancer patients should increase their protein intake.	253 (30.56)	501 (60.51)	74 (8.94)
7. Gastric cancer patients should ensure adequate daily water intake.	567 (68.48)	216 (26.09)	45 (5.43)
8. For gastric cancer patients who can eat orally, independent oral intake should be encouraged as the first priority.	284 (34.3)	481 (58.09)	63 (7.61)
9. When regular food intake cannot meet nutritional needs, additional nutritional supplement formulations can be used.	99 (11.96)	651 (78.62)	78 (9.42)
10. As treatment may affect appetite, patients can adopt a dietary pattern of frequent small meals.	277 (33.45)	521 (62.92)	30 (3.62)
11. During treatment, easily digestible foods are recommended, while hard, spicy, and irritating foods should be avoided.	640 (77.29)	179 (21.62)	9 (1.09)
12. Long-term consumption of spicy, pickled, and greasy foods is associated with an increased risk of gastric cancer.	641 (77.42)	170 (20.53)	17 (2.05)
13. Once malnutrition develops in gastric cancer patients, it can reduce the effectiveness of treatments like surgery, radiotherapy, and chemotherapy, and increase the likelihood of adverse effects.	166 (20.05)	615 (74.28)	47 (5.68)

In the attitude dimension, participants generally exhibited positive perceptions toward nutritional care, with 97.9% agreeing that nutrition management during treatment is essential for recovery and 95.2% expressing willingness to follow medical dietary advice. However, less favorable attitudes were observed in certain aspects: only 14.7% strongly agreed to accept parenteral nutrition if needed, 16.6% felt they had fully adapted to new eating patterns after diagnosis, and 51.3% strongly agreed to undergo regular nutritional assessments, indicating potential ambivalence toward active nutritional engagement (Table 3).

**TABLE 3:** Attitudes assessment.

Attitudes items, *n* (%)	Strongly agree	Agree	Neutral	Disagree	Strongly disagree
1. My cancer diagnosis and subsequent treatments have significantly affected my normal eating habits.	242 (29.23)	571 (68.96)	14 (1.69)	1 (0.12)	0 (0)
2. I am often concerned that I might be malnourished.	604 (72.95)	213 (25.72)	11 (1.33)	0 (0)	0 (0)
3. I believe that additional nutritional supplementation is necessary for most gastric cancer patients.	283 (34.18)	521 (62.92)	24 (2.9)	0 (0)	0 (0)
4. I consider nutritional management during treatment to be very important for recovery.	433 (52.29)	378 (45.65)	17 (2.05)	0 (0)	0 (0)
5. I am willing to undergo regular nutritional assessments.	425 (51.33)	363 (43.84)	40 (4.83)	0 (0)	0 (0)
6. I am willing to follow the dietary advice provided by my doctor or dietitian.	560 (67.63)	254 (30.68)	14 (1.69)	0 (0)	0 (0)
7. If nutritional needs cannot be met by diet or enteral nutrition alone, I am willing to accept parenteral nutrition.	122 (14.73)	647 (78.14)	59 (7.13)	0 (0)	0 (0)
8. Since my diagnosis, I have become accustomed to new eating patterns.	137 (16.55)	502 (60.63)	189 (22.83)	0 (0)	0 (0)
9. I am concerned that nutritional management could be too burdensome for my family.	502 (60.63)	304 (36.71)	22 (2.66)	0 (0)	0 (0)
10. I am worried that the cost of nutritional formulas might be high, creating a financial burden.	566 (68.36)	249 (30.07)	13 (1.57)	0 (0)	0 (0)

In the practice dimension, several behaviors were notably unsatisfactory. Only 6.4% of participants “always” monitored their body weight, 12.0% consistently ensured a balanced diet with adequate protein, and 6.3% regularly practiced eating frequent small meals. The main self-reported barriers to effective nutritional management included lack of knowledge (90.7%), insufficient time or overly complex guidelines (65.8%), and financial burden (45.3%) (P7). The most common information sources (P8) were attending physicians (93.5%), hospital lectures (66.8%), and new media platforms (58.0%) (Table 4).

**TABLE 4:** Practices assessment.

Practices items, *n* (%)	Always	Often	Sometimes	Seldom	Never
1. I adhere to the standard treatment regimen for gastric cancer as prescribed by my doctor.	218 (26.33)	543 (65.58)	67 (8.09)	0 (0)	0 (0)
2. In my daily life, I pay attention to ensuring adequate nutrient intake, such as by diversifying my food choices and guaranteeing the consumption of protein-rich foods.	99 (11.96)	510 (61.59)	216 (26.09)	3 (0.36)	0 (0)
3. I monitor my body weight regularly and stay aware of physical changes.	53 (6.4)	278 (33.57)	483 (58.33)	14 (1.69)	0 (0)
4. I adjust my diet based on recommendations from my doctor or dietitian.	164 (19.81)	534 (64.49)	130 (15.7)		0 (0)
5. I practice eating frequent small meals.	52 (6.28)	448 (54.11)	323 (39.01)	5 (0.6)	0 (0)
6. I choose soft, easily digestible foods and avoid those that are greasy, spicy, or hard to digest.	402 (48.55)	356 (43)	67 (8.09)	3 (0.36)	0 (0)

### Correlations among knowledge, attitudes, and practices

Correlation analyses among the three KAP domains (Supplementary Table 1) showed that all pairwise associations were statistically significant and positive, with knowledge positively correlated with attitudes (*r* = 0.284, *P* < 0.001) and practices (*r* = 0.163, *P* < 0.001), and attitudes positively correlated with practices (*r* = 0.230, *P* < 0.001).

### Path analysis

**FIGURE 1:**
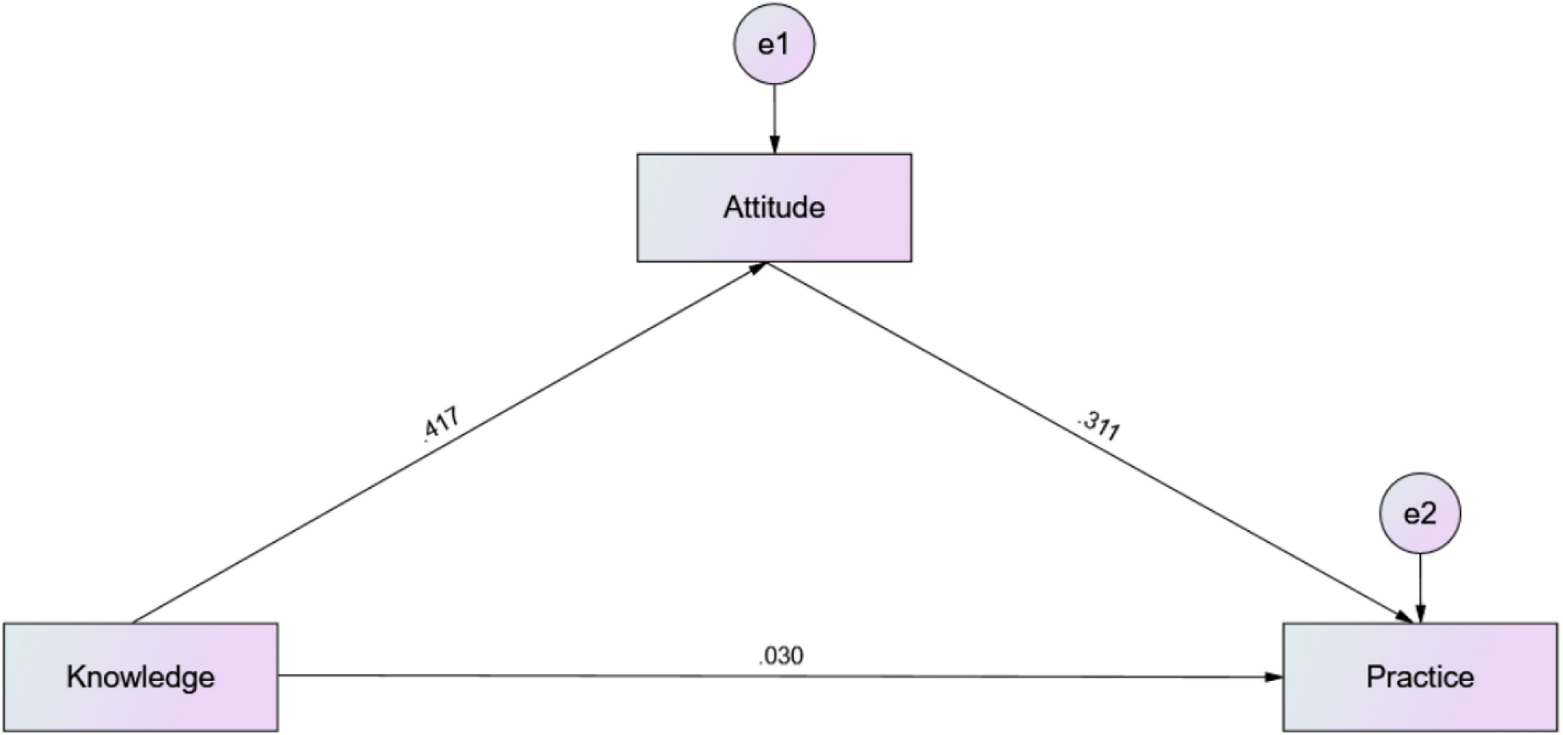
Exploratory path analysis model depicting the relationships among perceived knowledge, attitudes, and practices.

Path analysis was used to examine the hypothesized mediation of attitudes between knowledge and practices (Figure 1 and Table 5). All standardized factor loadings were statistically significant (*P* < 0.05), supporting the construct validity of the instrument. Perceived knowledge showed a significant positive association with attitudes (β = 0.417, 95% CI: 0.311–0.490, *P* = 0.019) and a total association with practices (β = 0.160, 95% CI: 0.076–0.241, *P* = 0.033), but its direct association with practices was not statistically significant (β = 0.030, 95% CI: −0.056 to 0.111, *P* = 0.655). Attitudes were significantly associated with practices (β = 0.311, 95% CI: 0.223–0.391, *P* = 0.007). The indirect association of perceived knowledge with practices through attitudes was significant (β = 0.130, 95% CI: 0.100–0.174, *P* = 0.004), confirming a potential mediating role of attitudes in the relationship between knowledge and practices.

**TABLE 5:** Exploratory path analysis.

Model paths	Standardized total effects		Standardized direct effects		Standardized indirect effects	
	β (95% CI)	*P*	β (95% CI)	*P*	β (95% CI)	*P*
Knowledge→attitudes	0.417 (0.311–0.490)	0.019	0.417 (0.311–0.490)	0.019	–	–
Knowledge→practices	0.160 (0.076–0.241)	0.033	0.030 (−0.056 to 0.111)	0.655	–	–
Attitudes→practices	0.311 (0.223–0.391)	0.007	0.311 (0.223–0.391)	0.007	–	–
Knowledge→practices	–	–	–	–	0.130 (0.100–0.174)	0.004

A multigroup analysis (MGA) was further conducted to evaluate whether the path model remained stable across subgroups defined by residential area, educational level, and monthly household income (Table 6). Notably, the direct conversion from knowledge to practice was significant only among urban patients (β = 0.168, *P* = 0.014), whereas it was non-significant for rural patients (β = −0.002, *P* = 0.957). Furthermore, education and income levels substantially modified the mediation pathways, indicating that socioeconomic disadvantages may hinder the efficient translation of nutritional knowledge into actual practice.

**TABLE 6:** Multigroup analysis of the exploratory path model across different demographic subgroups.

Characteristics	Model paths	Standardized total effects		Standardized direct effects		Standardized indirect effects	
		β (95% CI)	*P*	β (95% CI)	*P*	β (95% CI)	*P*
Residence Urban	Knowledge→attitude	0.879 (0.835, 0.904)	0.025	0.879 (0.835, 0.904)	0.025	–	–
	Knowledge→practice	0.877 (0.846, 0.900)	0.015	0.168 (0.109, 0.219)	0.014	–	–
	Attitude→practice	0.807 (0.763, 0.869)	0.004	0.807 (0.763, 0.869)	0.004	–	–
	Knowledge→practice	–	–	–	–	0.709 (0.657, 0.776)	0.005
Residence Rural	Knowledge→attitude	0.155 (0.022, 0.337)	0.094	0.155 (0.022, 0.337)	0.094	–	–
	Knowledge→practice	0.042 (−0.038, 0.168)	0.296	−0.002 (−0.083, 0.094)	0.957	–	–
	Attitude→practice	0.288 (0.204, 0.371)	0.007	0.288 (0.204, 0.371)	0.007	–	–
	Knowledge→practice	–	–	–	–	0.045 (0.010, 0.094)	0.072
Educational level Junior high school or below	Knowledge→attitude	0.566 (0.498, 0.642)	0.005	0.566 (0.498, 0.642)	0.005	–	–
	Knowledge→practice	0.313 (0.193, 0.381)	0.032	0.184 (0.059, .269)	0.028	–	–
	Attitude→practice	0.228 (0.130, 0.316)	0.010	0.228 (0.130, 0.316)	0.010	–	–
	Knowledge→practice	–	–	–	–	0.129 (0.074, 0.179)	0.012
Educational level Senior high school / Secondary specialized school	Knowledge→attitude	0.497 (0.363, 0.611)	0.009	0.497 (0.363, 0.611)	0.009	–	–
	Knowledge→practice	0.330 (0.208, 0.448)	0.012	0.256 (0.121, 0.399)	0.012	–	–
	Attitude→practice	0.149 (0.017, 0.254)	0.055	0.149 (0.017, 0.254)	0.055	–	–
	Knowledge→practice	–	–	–	–	0.074 (0.020,0.139)	0.025
Educational level Junior college (associate degree) or above	Knowledge→attitude	−0.156 (−0.374, 0.268)	0.733	−0.156 (−0.374, 0.268)	0.733	–	–
	Knowledge→practice	−0.258 (−0.429, −0.089)	0.016	−0.195 (−0.397, −0.005)	0.095	–	–
	Attitude→practice	0.408 (0.213, 0.532)	0.026	0.408 (0.213, 0.532)	0.026	–	–
	Knowledge→practice	–	–	–	–	−0.064 (−0.194, 0.066)	0.566
Monthly household income <2,000	Knowledge→attitude	0.630 (0.543, 0.695)	0.010	0.630 (0.543, 0.695)	0.010	–	–
	Knowledge→practice	0.355 (0.251, 0.468)	0.013	0.273 (0.138, 0.447)	0.007	–	–
	Attitude→practice	0.130 (−0.009, 0.248)	0.143	0.130 (−0.009, 0.248)	0.143	–	–
	Knowledge→practice	–	–	–	–	0.082 (−0.004, 0.156)	0.130
Monthly household income 2,000–5,000	Knowledge→attitude	0.393 (0.230, 0.493)	0.013	0.393 (0.230, 0.493)	0.013	–	–
	Knowledge→practice	0.205 (0.080, 0.314)	0.012	0.107 (−0.008, 0.229)	0.119	–	–
	Attitude→practice	0.249 (0.145, 0.344)	0.010	0.249 (0.145, 0.344)	0.010	–	–
	Knowledge→practice	–	–	–	–	0.098 (0.060, 0.151)	0.004
Monthly household income >5,000	Knowledge→attitude	0.105 (−0.169, 0.316)	0.528	0.105 (−0.169, 0.316)	0.528	–	–
	Knowledge→practice	−0.116 (−0.310, 0.038)	0.165	−0.161 (−0.318, −0.010)	0.078	–	–
	Attitude→practice	0.436 (0.285, 0.597)	0.007	0.436 (0.285, 0.597)	0.007	–	–
	Knowledge→practice	–	–	–	–	0.046 (−0.058, 0.138)	0.435

## Discussion

This study provides a comprehensive assessment of KAP concerning nutritional management among GC patients in China. The findings reveal a pronounced imbalance between patients’ willingness and their actual engagement in evidence-based nutritional behaviors, characterized by moderate attitudes but low knowledge and practice levels. Clinically, profiling patients’ KAP allows healthcare providers to identify key cognitive and behavioral obstacles that may compromise day-to-day nutritional management, and support more individualized nutritional counseling within routine care.

Similar to previous KAP investigations among GC populations (17), a “high attitude–low practice” pattern persists, reflecting a systemic gap in patient education and care integration. Perceived knowledge demonstrated both direct and indirect associations with practice, potentially mediated through attitudes, consistent with the classical KAP model observed across chronic disease and cancer populations (18–21). This relationship highlights that patients’ willingness alone is insufficient to drive behavior change unless supported by adequate disease-specific knowledge and practical skills. Moreover, the limited translation of positive attitudes into actionable behaviors suggests structural barriers—such as inadequate access to nutritional support services, insufficient communication between clinicians and dietitians, and inconsistent reinforcement of dietary recommendations across treatment stages. As evidenced by our data, 45.3% of patients expressed concerns regarding the financial burden of nutritional formulas. Furthermore, the lack of robust family support systems and the absence of systematic post-discharge follow-up act as substantial structural barriers, severely restricting the translation of positive nutritional attitudes into actionable behaviors. These findings underscore the necessity of embedding structured nutritional counseling into the routine management of GC, integrating multidisciplinary input, and providing patients with actionable, stage-specific guidance that can bridge the gap between motivation and implementation.

Differences in KAP scores were observed across groups with varying educational attainment and household income, indicating that socioeconomic factors are associated with disparities in nutritional knowledge, attitudes, and practices. This aligns with nationwide data from the China Precision Nutrition and Health-KAP study (16), which emphasized the impact of health education inequality on clinical outcomes. Furthermore, participants residing in urban areas demonstrated higher KAP scores compared with their rural counterparts, consistent with caregiver-based studies reporting limited access to nutritional counseling and dietitian support in rural settings (22). These findings reinforce that despite advances in multidisciplinary cancer care, the translation of nutritional knowledge into consistent practice remains insufficient. Nutritional management should therefore be recognized not merely as an adjunct therapy, but as a core component of comprehensive GC care that directly influences survival and quality of life (9, 23).

The persistently low knowledge level among GC patients underscores a deep-seated gap in nutritional education within oncology services. Most participants were aware that diet impacts recovery but lacked understanding of the mechanisms or guidelines for protein, micronutrient, and energy intake after surgery or chemotherapy. Similar deficits were reported by Yu et al. who found that only 37.5% of GC patients in Wuxi demonstrated adequate awareness of nutrition therapy (17), and by Qu et al. who showed that even family caregivers exhibited poor comprehension of basic nutritional principles (24). This lack of knowledge is partly attributable to clinical workflow constraints, where oncologic treatment efficacy often overshadows nutritional counseling during patient education (25). Additionally, standardized educational frameworks for nutritional rehabilitation are lacking in many Chinese cancer centers. Even when education occurs, it is often verbal, unstructured, and insufficiently reinforced through written or visual aids (26). Internationally, structured nutrition education based on behavioral models, such as the Health Belief Model, has significantly improved dietary practices among at-risk populations (27). The integration of visual learning materials, digital monitoring, and repeated follow-ups has been shown to enhance both comprehension and adherence. However, such programs remain scarce in China, especially in rural hospitals lacking dietitians or digital infrastructure. Therefore, implementing a multimodal, longitudinal education model—spanning preoperative counseling, inpatient reinforcement, and post-discharge digital follow-up—should be prioritized. Similar multidisciplinary approaches have proven effective in improving postoperative recovery, reducing feeding intolerance, and enhancing quality of life in GC patients (9, 28).

From the perspective of attitude, the present study revealed that most GC patients expressed positive beliefs regarding the importance of nutritional management. This finding aligns with recent evidence from both patient- and caregiver-based studies showing that while attitude exhibits a significant positive association with nutritional practice, the effect is often weakened without structured behavioral reinforcement and environmental support (17). Similarly, a cross-sectional study of 508 caregivers found that higher attitude scores were independently associated with improved dietary management practices after gastrectomy, underscoring the mediating role of attitude in nutritional compliance (22). Broader evidence from cancer survivorship research also confirms that patients with more positive dietary attitudes exhibit greater engagement in nutritional self-care and adherence to medical guidance (29). Collectively, these findings highlight the need for sustained, motivation-oriented education strategies—integrating psychological support and self-efficacy enhancement—to effectively convert positive attitudes into tangible, health-promoting nutritional practices.

Despite generally positive attitudes toward nutritional management, most patients failed to translate these attitudes into practice. This “attitude–practice gap” parallels observations from chronic disease management and cancer survivorship studies (30). While over 70% of respondents expressed strong agreement on the importance of diet during treatment, less than half reported consistent adherence to dietary advice or use of nutritional supplements. Barriers to practice include limited guidance from clinicians, financial constraints, and psychosocial challenges such as fear of symptom worsening or uncertainty about dietary recommendations. Wu et al. similarly noted that colorectal cancer patients undergoing radiotherapy exhibited positive beliefs but low adherence to nutritional behavior change (31). Attitude alone, therefore, is insufficient—practical skills, confidence, and environmental reinforcement are necessary to sustain behavior. To address this gap, behaviorally oriented interventions—combining counseling, digital health support, and peer education—should be implemented. Evidence from oncology and hemodialysis populations shows that combining education with real-time feedback, goal setting, and family involvement significantly improves nutritional behavior and patient satisfaction (32).

The present findings have several implications for clinical oncology nutrition practice. First, standardized screening for malnutrition risk and KAP status should be integrated into routine GC management, using validated tools and questionnaires with proven psychometric properties (12). Second, patient education should shift from a passive, one-time model to an active, continuous, and skill-based framework supported by digital health tools (33, 34). Targeted educational interventions, potentially leveraging digital health tools such as mobile applications, online platforms, or telehealth services, could be instrumental in addressing identified gaps and improving both knowledge and practical engagement. Third, multidisciplinary collaboration—among surgeons, oncologists, nurses, dietitians, and psychologists—should be institutionalized to ensure comprehensive nutritional rehabilitation (9). Fourth, caregivers’ inclusion in educational interventions is critical, as caregiver KAP directly influences patients’ adherence and recovery (35). From a policy perspective, it is essential that nutritional management be explicitly incorporated into national GC guidelines, with reimbursement mechanisms for professional nutritional counseling, oral supplements, and enteral feeding support. In developed countries, multidisciplinary nutrition pathways have been shown to reduce hospital stay and readmission rates (16). China could adapt such models, leveraging its expanding digital health infrastructure to scale educational outreach to underserved populations.

This study’s strengths include its large sample size, psychometrically validated instrument, and exploratory path analysis that elucidated both direct and mediating pathways between KAP domains. However, several limitations must be acknowledged. First, the cross-sectional design limits causal inference. Second, self-reported data may be subject to bias; notably, objective nutritional assessment tools such as NRS-2002 or PG-SGA were excluded during the pilot phase due to the difficulty patients experienced in accurately self-reporting complex clinical criteria (e.g., specific weight loss percentages or changes in dietary intake volumes). In addition, two reverse-scored attitude items (A9 and A10) showed slightly negative corrected item-total correlations, suggesting that some participants may not have correctly identified the reverse-worded phrasing; this reflects an inherent limitation of self-administered reverse-scored items that cannot be entirely avoided. Consequently, the reliance on self-reported BMI without the incorporation of objective clinical nutritional biomarkers (e.g., serum albumin levels) prevents a holistic clinical correlation between KAP scores and actual nutritional status or clinical outcomes. Third, the lack of stratified analyses according to specific treatment phases (e.g., newly diagnosed, perioperative, chemotherapy) restricts the evaluation of stage-specific nutritional needs. Fourth, regional sampling may restrict generalizability. Fifth, while the study focused on patients, caregiver and healthcare provider perspectives were not directly examined—both of which significantly influence nutrition outcomes. Sixth, recurrence status and detailed metastatic characteristics were not separately collected, and stage IV disease could not distinguish newly diagnosed metastatic disease from recurrent disease. Additionally, the absence of qualitative data, such as interviews or focus group discussions, restricts a deeper exploration of the cultural and psychological factors affecting patient behaviors. Future research should prioritize longitudinal and interventional study designs—such as randomized controlled trials assessing digital education platforms or multidisciplinary nutrition clinics—to establish causality, evaluate the long-term effectiveness of educational programs, and directly assess their impact on clinical outcomes like treatment tolerance and survival.

## Conclusion

In conclusion, despite exhibiting positive attitudes toward nutritional management, GC patients demonstrate significant gaps in nutritional knowledge and practical implementation, a discrepancy strongly moderated by socioeconomic factors such as residence and education. This study underscores that motivation alone is insufficient without structural support. Future studies should evaluate whether multidisciplinary, skill-based nutritional counseling and digital health tools can help address the observed knowledge–practice gaps.

## Data Availability

The original contributions presented in this study are included in the article/Supplementary material, further inquiries can be directed to the corresponding author.

## Footnotes

1
https://www.wjx.cn/

## Supplementary material

Supplementary Figure 1 Confirmatory factor analysis (CFA) model for the knowledge, attitudes, and practices (KAP) questionnaire on nutritional management among gastric cancer patients.

Supplementary Figure 2 Participant flow diagram.
