# Supplementary material for: Perceived knowledge, attitudes, and practices regarding nutritional management among gastric cancer patients: an exploratory path analysis
Source: Front Nutr. 2026 Aug 5;13:1766768. doi: 10.3389/fnut.2026.1766768 (PMC13485532; doi:10.3389/fnut.2026.1766768)
Supplement: Supplementary file 1 [file Table_1.docx]

**Supplementary Table S1.** Correlation Analysis

|  | Knowledge | Attitudes | Practices |
| --- | --- | --- | --- |
| Knowledge | 1 |  |  |
| Attitudes | 0.284 (P<0.001) | 1 |  |
| Practices | 0.163 (P<0.001) | 0.230 (P<0.001) | 1 |

**Supplementary Table S2.** Item-level factor loadings and psychometric parameters from the Confirmatory Factor Analysis (CFA) for the KAP questionnaire.

|  |  |  | **Estimate** | **Standardized Estimate** | **S.E.** | **C.R.** | **P** |
| --- | --- | --- | --- | --- | --- | --- | --- |
| K1 | <--- | Knowledge | 1.000 | 0.701 |  |  |  |
| K2 | <--- | Knowledge | 1.029 | 0.714 | 0.052 | 19.920 | <0.001 |
| K3 | <--- | Knowledge | 0.517 | 0.400 | 0.050 | 10.245 | <0.001 |
| K4 | <--- | Knowledge | 0.701 | 0.525 | 0.053 | 13.161 | <0.001 |
| K5 | <--- | Knowledge | 0.330 | 0.264 | 0.048 | 6.894 | <0.001 |
| K6 | <--- | Knowledge | 0.725 | 0.518 | 0.056 | 12.921 | <0.001 |
| K7 | <--- | Knowledge | 0.843 | 0.615 | 0.062 | 13.625 | <0.001 |
| K8 | <--- | Knowledge | 0.709 | 0.514 | 0.060 | 11.875 | <0.001 |
| K9 | <--- | Knowledge | 0.503 | 0.464 | 0.042 | 11.958 | <0.001 |
| K10 | <--- | Knowledge | 0.615 | 0.491 | 0.050 | 12.358 | <0.001 |
| K11 | <--- | Knowledge | 0.580 | 0.546 | 0.042 | 13.680 | <0.001 |
| K12 | <--- | Knowledge | 0.615 | 0.548 | 0.045 | 13.791 | <0.001 |
| K13 | <--- | Knowledge | 0.316 | 0.274 | 0.043 | 7.301 | <0.001 |
| A1 | <--- | Attitude | 1.000 | 0.246 |  |  |  |
| A2 | <--- | Attitude | 1.783 | 0.450 | 0.276 | 6.465 | <0.001 |
| A3 | <--- | Attitude | 0.967 | 0.225 | 0.198 | 4.897 | <0.001 |
| A4 | <--- | Attitude | 0.784 | 0.177 | 0.185 | 4.249 | <0.001 |
| A5 | <--- | Attitude | 1.653 | 0.345 | 0.278 | 5.954 | <0.001 |
| A6 | <--- | Attitude | 1.414 | 0.337 | 0.239 | 5.923 | <0.001 |
| A7 | <--- | Attitude | 0.670 | 0.177 | 0.158 | 4.234 | <0.001 |
| A8 | <--- | Attitude | 0.468 | 0.090 | 0.191 | 2.449 | 0.014 |
| A9 | <--- | Attitude | -0.302 | -0.067 | 0.160 | -1.887 | 0.059 |
| A10 | <--- | Attitude | -0.189 | -0.046 | 0.147 | -1.286 | 0.199 |
| P1 | <--- | Practice | 1.000 | 0.400 |  |  |  |
| P2 | <--- | Practice | 0.855 | 0.313 | 0.158 | 5.395 | <0.001 |
| P3 | <--- | Practice | 0.533 | 0.186 | 0.163 | 3.279 | 0.001 |
| P4 | <--- | Practice | 0.995 | 0.373 | 0.194 | 5.122 | <0.001 |
| P5 | <--- | Practice | 0.503 | 0.187 | 0.138 | 3.640 | <0.001 |
| P6 | <--- | Practice | 1.472 | 0.514 | 0.252 | 5.837 | <0.001 |

**Supplementary Table S3.** Item-total correlations and Cronbach’s α-if-item-deleted values for the overall KAP questionnaire based on the full study cohort

|  | **Scale Mean if Item Deleted** | **Scale Variance if Item Deleted** | **Corrected Item-Total Correlation** | **Cronbach's Alpha if Item Deleted** |
| --- | --- | --- | --- | --- |
| K1 | 82.87 | 31.461 | 0.472 | 0.757 |
| K2 | 82.64 | 30.846 | 0.561 | 0.751 |
| K3 | 83.51 | 31.975 | 0.447 | 0.759 |
| K4 | 82.98 | 32.178 | 0.395 | 0.761 |
| K5 | 83.85 | 33.430 | 0.217 | 0.770 |
| K6 | 83.03 | 31.884 | 0.421 | 0.760 |
| K7 | 82.62 | 31.623 | 0.468 | 0.757 |
| K8 | 82.98 | 31.945 | 0.412 | 0.760 |
| K9 | 83.22 | 32.482 | 0.446 | 0.760 |
| K10 | 82.95 | 32.579 | 0.360 | 0.764 |
| K11 | 82.48 | 32.516 | 0.452 | 0.760 |
| K12 | 82.49 | 32.608 | 0.406 | 0.762 |
| K13 | 83.10 | 33.338 | 0.261 | 0.768 |
| A1 | 79.97 | 33.358 | 0.255 | 0.769 |
| A2 | 79.53 | 32.448 | 0.433 | 0.761 |
| A3 | 79.93 | 33.117 | 0.275 | 0.768 |
| A4 | 79.74 | 33.917 | 0.133 | 0.775 |
| A5 | 79.78 | 32.701 | 0.297 | 0.767 |
| A6 | 79.59 | 32.976 | 0.309 | 0.766 |
| A7 | 80.17 | 33.612 | 0.227 | 0.770 |
| A8 | 80.31 | 33.551 | 0.152 | 0.775 |
| A9 | 79.67 | 34.917 | -0.027 | 0.782 |
| A10 | 79.58 | 34.839 | -0.009 | 0.780 |
| P1 | 80.06 | 33.385 | 0.209 | 0.771 |
| P2 | 80.39 | 33.833 | 0.118 | 0.776 |
| P3 | 80.80 | 34.296 | 0.045 | 0.781 |
| P4 | 80.21 | 32.944 | 0.255 | 0.769 |
| P5 | 80.59 | 33.919 | 0.109 | 0.777 |
| P6 | 79.85 | 32.334 | 0.309 | 0.766 |
