# Supplementary material for: Perceived knowledge, attitudes, and practices regarding nutritional management among gastric cancer patients: an exploratory path analysis
Source: Front Nutr. 2026 Aug 5;13:1766768. doi: 10.3389/fnut.2026.1766768 (PMC13485532; doi:10.3389/fnut.2026.1766768)
Supplement: Supplementary file 2 [file Table_2.doc]

|  | **Questionnaire Number:** | | | |
| --- | --- | --- | --- | --- |
|  | To the participant:  We are healthcare professionals from Shanxi Cancer Hospital. You are cordially invited to participate in our research study. This study aims to understand the knowledge, attitudes, and practices regarding nutritional management among gastric cancer patients. The findings will provide a basis for developing scientific intervention strategies, which may benefit future patients and help improve health outcomes. Your participation is entirely voluntary. This study has been reviewed and approved by the Ethics Committee.  If you agree to participate, please note the following:   1) You will be asked to complete a questionnaire. There are no right or wrong answers; please respond based on your actual situation. Feel free to ask us any questions during the process. Please submit the questionnaire upon completion. 2) This is a simple survey and will not cause harm to your physical or psychological well-being. The questionnaire includes questions of a personal nature (e.g., gender, age). All information provided will be kept strictly confidential. 3) As a participant, you have the right to inquire about information related to this study. Should you decide to withdraw at any time, please inform us, and your data will not be included in the study results.   Thank you sincerely for taking the time to support our research.  □I am aware of and agree to use the collected data for scientific research.  Informed consent signature：  Date： Year Month Day | | | |
| **Part one General Information** | | | |  |
| **1. Gender:** | | a. Male  b. Female | |  |
| **2. Age:** | | | |  |
| **3.** **Area of residence** | | a. Rural  b. Urban | |  |
| **4. Educational level** | | a. Junior high school or below  b. Senior high school / Secondary specialized school  c. Junior college  d. Bachelor or above | |  |
| **5. Marital status** | | a. Unmarried  b. Married  c. Divorced  d. Widowed | |  |
| **6. Monthly household income: ______Yuan** | | a. <2000  b. 2000-5000  c. 5000-10000  d. 10000-20000  e. >20000 | |  |
| **7.** **Types of medical insurance** | | a. Urban Employee Basic Medical Insurance  b. New Rural Cooperative Medical Insurance  c. Urban Resident Basic Medical Insurance  d. Medical Insurance for Retired Veterans/Officials  e. Commercial Health Insurance  f. No Medical Insurance | |  |
| **8. Gastric cancer staging** | | a. I stage b. II stage c. III stage d. IV stage | |  |
| **9. Time since diagnosis** | | a. < 1 month  b. 1-3 months  c. 3-6 months  d. 6 months - 1 year  e.1-3 year  f. >3 years | |  |
| **10．Have you undergone treatment? (Select all that apply)** | | | a. Treatment not yet initiated  b. Surgery  c. Radiotherapy  d. Chemotherapy  e. Targeted therapy  f. Immunotherapy |  |
| **11.** **What is your current height? (cm)（Please enter your height in centimeters (cm) as a whole number）** | | | |  |
| **12. What is your current weight? (kg)（Please enter your height in centimeters (kg) as a whole number）** | | | |  |
| **13. Recent body weight change** | | | a. No significant change  b. Approximately 5% weight loss over the past 3 months  c. Approximately 5% weight loss over the past 2 months  d. Approximately 5% weight loss over the past month |  |
| **14. Recent Change in Food Intake (over the past week)** | | | a. >25% Food intake decreased by >25%  b. Food intake decreased by >50%  c. Food intake decreased by >75%  d. No significant change |  |
| **15. Concomitant Diseases (select all that apply)** | | | a. Hematologic malignancy b. Severe pneumonia c. Major abdominal surgery d. Stroke e. Bone marrow transplantation f. None of the above |  |
| **16. Time spent on physical activity per day** | | | a. 0 hour b. 0-1 hour c. 1-2 hours d. > 2 hours |  |
| **17. Sleep quality** | | | a. Good b. Poor (frequent insomnia or night awakening) |  |
| **18. History of gastric cancer in first-degree relatives?** | | | a. Yes b. No |  |
| **19. History of smoking** | | | a. Never b. Occasionally c. Frequently d. Heavily |  |
| **20. History of drinking** | | | a. Never b. Occasionally c. Frequently d. Heavily |  |
| **21. Dietary habits** | | | a. Preferentially light b. Preferentially spicy c. Preferentially greasy |  |
| **22. Have you ever received nutritional education from a hospital or other healthcare institution?** | | | a. Yes  b. No |  |

| **Part two. Knowledge assessment** | | | |  |
| --- | --- | --- | --- | --- |
| **Please indicate your level of familiarity with the following statements:** | | | | |
| 1. **Gastric cancer patients are highly susceptible to malnutrition** | a. Well understood | b. Heard of | c. Unclear | |
| 2. **Nutritional management is essential for gastric cancer patients throughout the entire treatment course** | a. Well understood | b. Heard of | c. Unclear | |
| 3. **Are you familiar with the Dietary Guidelines for Chinese Residents** | a. Well understood | b. Heard of | c. Unclear | |
| 4. **Gastric cancer patients should maintain a diverse diet and strive for nutritional balance** | a. Well understood | b. Heard of | c. Unclear | |
| 5. **The estimated daily energy requirement for cancer patients is approximately 20-30 kcal per kg of body weight.** | a. Well understood | b. Heard of | c. Unclear | |
| 6. **Compared to healthy individuals, gastric cancer patients should increase their protein intake.** | a. Well understood | b. Heard of | c. Unclear | |
| 7. **Gastric cancer patients should ensure adequate daily water intake.** | a. Well understood | b. Heard of | c. Unclear | |
| 8. **For gastric cancer patients who can eat orally, independent oral intake should be encouraged as the first priority.** | a. Well understood | b. Heard of | c. Unclear | |
| 9. **When regular food intake cannot meet nutritional needs, additional nutritional supplement formulations can be used.** | a. Well understood | b. Heard of | c. Unclear | |
| 10. **As treatment may affect appetite, patients can adopt a dietary pattern of frequent small meals.** | a. Well understood | b. Heard of | c. Unclear | |
| 11. **During treatment, easily digestible foods are recommended, while hard, spicy, and irritating foods should be avoided.** | a. Well understood | b. Heard of | c. Unclear | |
| 12. **Long-term consumption of spicy, pickled, and greasy foods is associated with an increased risk of gastric cancer.** | a. Well understood | b. Heard of | c. Unclear | |
| 13. **Once malnutrition develops in gastric cancer patients, it can reduce the effectiveness of treatments like surgery, radiotherapy, and chemotherapy, and increase the likelihood of adverse effects.** | a. Well understood | b. Heard of | c. Unclear | |
| 14. **The Han ethnic group has the smallest population among China's 56 ethnic groups.** | a. True | b. Wrong | c. Unclear | |

| **Part three. Attitudes assessment** | | | | | |
| --- | --- | --- | --- | --- | --- |
| **1. My cancer diagnosis and subsequent treatments have significantly affected my normal eating habits.** | a. Strongly agree | b. Agree | c. Neutral | d. Disagree | e. Strongly disagree |
| **2. I am often concerned that I might be malnourished.** | a. Strongly agree | b. Agree | c. Neutral | d. Disagree | e. Strongly disagree |
| **3. I believe that additional nutritional supplementation is necessary for most gastric cancer patients.** | a. Strongly agree | b. Agree | c. Neutral | d. Disagree | e. Strongly disagree |
| **4. I consider nutritional management during treatment to be very important for recovery.** | a. Strongly agree | b. Agree | c. Neutral | d. Disagree | e. Strongly disagree |
| **5. I am willing to undergo regular nutritional assessments.** | a. Strongly agree | b. Agree | c. Neutral | d. Disagree | e. Strongly disagree |
| **6. I am willing to follow the dietary advice provided by my doctor or dietitian.** | a. Strongly agree | b. Agree | c. Neutral | d. Disagree | e. Strongly disagree |
| **7.** **If nutritional needs cannot be met by diet or enteral nutrition alone, I am willing to accept parenteral nutrition.** | a. Strongly agree | b. Agree | c. Neutral | d. Disagree | e. Strongly disagree |
| **8. Since my diagnosis, I have become accustomed to new eating patterns.** | a. Strongly agree | b. Agree | c. Neutral | d. Disagree | e. Strongly disagree |
| **9. I am concerned that nutritional management could be too burdensome for my family.** | a. Strongly agree | b. Agree | c. Neutral | d. Disagree | e. Strongly disagree |
| **10. I am worried that the cost of nutritional formulas might be high, creating a financial burden.** | a. Strongly agree | b. Agree | c. Neutral | d. Disagree | e. Strongly disagree |

| **Part four. Practices assessment**  **Always:** Able to comply almost 100% of the time  **Often:** Able to comply approximately >70% of the time  **Sometimes:** Able to comply approximately 40-70% of the time  **Seldom:** Able to comply approximately 10-40% of the time  **Never:** Unable to comply or able to comply <10% of the time | | | | | |
| --- | --- | --- | --- | --- | --- |
| **1. I adhere to the standard treatment regimen for gastric cancer as prescribed by my doctor.** | a.总是 | b.经常 | c.有时 | d.很少 | e.从不 |
| **2. In my daily life, I pay attention to ensuring adequate nutrient intake, such as by diversifying my food choices and guaranteeing the consumption of protein-rich foods.** | a.总是 | b.经常 | c.有时 | d.很少 | e.从不 |
| **3. I monitor my body weight regularly and stay aware of physical changes.** | a.总是 | b.经常 | c.有时 | d.很少 | e.从不 |
| **4. I adjust my diet based on recommendations from my doctor or dietitian.** | a.总是 | b.经常 | c.有时 | d.很少 | e.从不 |
| **5. I practice eating frequent small meals.** | a.总是 | b.经常 | c.有时 | d.很少 | e.从不 |
| **6. I choose soft, easily digestible foods and avoid those that are greasy, spicy, or hard to digest.** | a.总是 | b.经常 | c.有时 | d.很少 | e.从不 |
| **7.** **Regarding your nutritional management during treatment, have you faced any of the following challenges? (Select all that apply)**  a. Poor appetite or indigestion, preventing normal eating  b. Lack of knowledge about nutritional management, unsure how to proceed  c. Nutritional management guidelines are too complex; insufficient time  d. Excessive financial burden | | | | | |
| **8. Through which channels have you acquired knowledge about nutritional management? (Select all that apply)**  a. Clinical dietitian  b. Attending physician or other doctors (patient education)  c. Hospital lectures and educational sessions  d. New media platforms (e.g., WeChat, Weibo, Douyin/TikTok, Xiaohongshu)  e. Traditional media (e.g., television, newspapers)  f. Relatives or friends | | | | | |

|  | 问卷编号： | | | | | | | |
| --- | --- | --- | --- | --- | --- | --- | --- | --- |
|  | 亲爱的参与者：  我们是山西肿瘤医院的医务人员，诚挚邀请您参与我们的课题研究。本研究旨在了解胃癌患者对于营养管理的知识、态度和实践，为制定科学的干预策略提供依据，这可能在未来帮助到更多的人，改善他们的健康情况。您参与这项研究是自愿的，本研究已通过伦理审查委员会的审查，如果您同意参与本研究，请参阅以下说明。  1.请您完成问卷，答案无关对错，您只需要根据实际情况填写。回答过程中的任何问题您都可以向我们提出，完成后，请您及时提交。  2.本研究是简单的问卷调查，不会对您的身体和心理状况造成伤害，但会涉及一些隐私问题，如您的性别、年龄等，我们会严格保密，不会泄露您的信息，请您放心填写。  3.作为参与者，您可以随时了解与本研究相关的信息和研究进展，如果您决定退出研究，请告知我们，您的数据将不包含在研究结果中。  最后，衷心感谢您能百忙之中抽出时间支持我们的科学研究！  □我已知晓并同意将所收集的数据用于科学研究。  知情同意签字：  参与时间： 年 月 日 | | | | | | | |
| **第一部分 基本信息** | | | | | | | |  |
| **1.您的性别：** | | a.男  b.女 | | | | | |  |
| **2.您的年龄： 。** | | | | | | | |  |
| **3.您的居住地：** | | a.农村  b.城市 | | | | | |  |
| **4.您的教育程度：** | | a.初中及以下  b.高中/中专  c.大专  d.本科及以上 | | | | | |  |
| **5.您的婚姻状况：** | | a.未婚  b.已婚  c.离异  d.丧偶 | | | | | |  |
| **6.您的家庭人均月收入为:______元** | | a.<2千  b.2千-5千  c.5千-1万  d.1万-2万  e.>2万 | | | | | |  |
| **7.** **您的医保类型：（多选）** | | a.城镇职工基本医疗保险  b.新农合医疗保险  c.城镇居民基本医疗保险  d.离休干部医疗保险  e.商业保险  f.无保险 | | | | | |  |
| **8.您的胃癌分期：** | | e. I期 f. II期 g. III期 h. IV期 | | | | | |  |
| **9. 您确诊胃癌的时间：** | | a.1月内  b.1-3月  c.3-6月  d.6月-1年  e.1-3年  f.3年以上 | | | | | |  |
| **10．您接受过以下哪些治疗（可多选）：** | | | a.未开始治疗  b.手术  c.放疗  d.化疗  e.靶向治疗  f.免疫治疗 | | | | |  |
| **11.您现在的身高： （厘米）（问卷星中请设置整数验证）** | | | | | | | |  |
| **12.您现在的体重： （公斤）（问卷星中请设置整数验证）** | | | | | | | |  |
| **13.近期您的体重变化：** | | | a.无变化  b.近3月体重下降5%  c.近2月体重下降5%  d.近1月体重下降5% | | | | |  |
| **14.近期您的饮食情况变化：** | | | a.近1周内进食量减少>25%  b.近1周内进食量减少>50%  c.近1周内进食量减少>75%  d.无变化 | | | | |  |
| **15.您是否合并以下疾病：** | | | g. 血液恶性肿瘤 h. 重症肺炎 i. 腹部大型手术 j. 脑卒中 k. 骨髓移植 l. 以上均无 | | | | |  |
| **16.您每天进行体育锻炼的时间：** | | | e. 0小时 f. 0-1小时 g. 1-2小时 h. 2小时以上 | | | | |  |
| **17.您目前的睡眠情况：** | | | c. 睡眠良好 d. 睡眠质量较差，经常失眠或惊醒 | | | | |  |
| **18.您的直系亲属中是否有胃癌患者：** | | | c. 是 d. 否 | | | | |  |
| **19.您是否抽烟：** | | | e. 从不 f. 偶尔 g. 经常 h. 大量 | | | | |  |
| **20.您是否饮酒：** | | | e. 从不 f. 偶尔 g. 经常 h. 大量 | | | | |  |
| **21.患病前您的饮食习惯** | | | d. 偏清淡 e. 偏辛辣 f. 偏油腻 | | | | |  |
| **22.您是否接受过医院或其他医疗机构对于营养管理的教育知识宣传：** | | | a.有  b.没有 | | | | |  |
| **第二部分 对营养管理的了解情况** | | | | | | |  | |
| **下列陈述，您了解的程度是：** | | | | | | | | |
| 15. **胃癌患者很容易出现营养不良。** | | | | a.很了解 | b.听说过 | c.不清楚 | | |
| 16. **胃癌患者在治疗全程均需注意的营养管理** | | | | a.很了解 | b.听说过 | c.不清楚 | | |
| 17. **您了解中国居民膳食指南吗？** | | | | a.很了解 | b.听说过 | c.不清楚 | | |
| 18. **胃癌患者应注意多样化饮食，尽量做到营养均衡。** | | | | a.很了解 | b.听说过 | c.不清楚 | | |
| 19. **肿瘤患者的能量需要量大约为20-30kcal/(kg·d)。** | | | | a.很了解 | b.听说过 | c.不清楚 | | |
| 20. **相较于正常人，胃癌患者应该额外增加蛋白质的摄入量。** | | | | a.很了解 | b.听说过 | c.不清楚 | | |
| 21. **胃癌患者应每天摄入足够的水。** | | | | a.很了解 | b.听说过 | c.不清楚 | | |
| 22. **对可以进食的胃癌患者，优先鼓励患者自主进食。** | | | | a.很了解 | b.听说过 | c.不清楚 | | |
| 23. **当正常的饮食不能满足患者的营养需求时，可采用额外的营养补充制剂。** | | | | a.很了解 | b.听说过 | c.不清楚 | | |
| 24. **患者在治疗期间的食欲会受到影响，可采用少食多餐的方式进食。** | | | | a.很了解 | b.听说过 | c.不清楚 | | |
| 25. **治疗期间应选择易消化的食物，避免坚硬辛辣刺激的食物。** | | | | a.很了解 | b.听说过 | c.不清楚 | | |
| 26. **辛辣、腌制、油腻食物的长期摄入和胃癌的发生有关。** | | | | a.很了解 | b.听说过 | c.不清楚 | | |
| 27. **胃癌患者发生营养不良后，手术、放疗和化疗等不同治疗措施疗效都会受影响，发生不良反应的几率会增加。** | | | | a.很了解 | b.听说过 | c.不清楚 | | |
| 28. **中国56个民族中汉族人口最少。** | | | | a.对 | b.错 | c.不清楚 | | |

| **第三部分 对营养管理的态度想法** | | | | | |
| --- | --- | --- | --- | --- | --- |
| **1.肿瘤和后续的治疗影响了我正常的饮食习惯。** | a.非常同意 | b.同意 | c.一般 | d.不同意 | e.非常不同意 |
| **2.我时常担心自己有营养不良的状况。** | a.非常同意 | b.同意 | c.一般 | d.不同意 | e.非常不同意 |
| **3.我认为对一般胃癌患者而言，额外的营养补充是必要的。** | a.非常同意 | b.同意 | c.一般 | d.不同意 | e.非常不同意 |
| **4.我认为治疗期间的营养管理对疾病康复很重要。** | a.非常同意 | b.同意 | c.一般 | d.不同意 | e.非常不同意 |
| **5.我愿意接受定期的营养评估。** | a.非常同意 | b.同意 | c.一般 | d.不同意 | e.非常不同意 |
| **6.我愿意按照医生或营养师的要求控制饮食。** | a.非常同意 | b.同意 | c.一般 | d.不同意 | e.非常不同意 |
| **7. 单纯依靠饮食、肠内营养不能满足营养需要时，我愿意采用肠外营养。** | a.非常同意 | b.同意 | c.一般 | d.不同意 | e.非常不同意 |
| **8.患病以来，我已经习惯了新的吃饭方式。** | a.非常同意 | b.同意 | c.一般 | d.不同意 | e.非常不同意 |
| **9.我担心营养管理过于麻烦，给家人造成负担。** | a.非常同意 | b.同意 | c.一般 | d.不同意 | e.非常不同意 |
| **10.我担心营养制剂价格较高，经济负担较重。** | a.非常同意 | b.同意 | c.一般 | d.不同意 | e.非常不同意 |

| **第四部分 对营养管理的行为实践**  总是：几乎100%能做到；经常：约能做到70%以上；有时：约能做到40-70%；很少：约能做到10-40%；从不：从来做不到或仅能做到10%以下 | | | | | |
| --- | --- | --- | --- | --- | --- |
| **1.我按医嘱接受胃癌的规范治疗。** | a.总是 | b.经常 | c.有时 | d.很少 | e.从不 |
| **2.我在日常生活中会注意加强营养充分的摄入，如食物多样化，保证蛋白类食物的摄入。** | a.总是 | b.经常 | c.有时 | d.很少 | e.从不 |
| **3.我定期测量体重，关注身体变化。** | a.总是 | b.经常 | c.有时 | d.很少 | e.从不 |
| **4.我会根据医生或营养师的建议来调整饮食。** | a.总是 | b.经常 | c.有时 | d.很少 | e.从不 |
| **5.我会注意少食多餐。** | a.总是 | b.经常 | c.有时 | d.很少 | e.从不 |
| **6.** **我会选择易于消化的软食，避免油腻、辛辣、难以消化的食物。** | a.总是 | b.经常 | c.有时 | d.很少 | e.从不 |
| **7.对于您在治疗期间的营养管理，您是否面临以下问题：（可多选）**  a.食欲不振或消化不良，无法正常饮食  b.对营养管理的知识了解不多，不知道该如何进行营养管理  c.营养管理的要求过于复杂，没有足够的时间  d.经济负担过重 | | | | | |
| **8.您了解营养管理的相关知识的途径？（可多选）**  a. 营养科医生  b. 主管医生或其他医生科普  c. 医院讲座和宣教  d.新媒体（微信、微博、抖音、小红书等）  e. 多媒体（电视、报纸等）  f.亲戚朋友 | | | | | |
